# Fungus-mediated green synthesis of nano-silver using *Aspergillus sydowii* and its antifungal/antiproliferative activities

**DOI:** 10.1038/s41598-021-89854-5

**Published:** 2021-05-14

**Authors:** Dongyang Wang, Baiji Xue, Lin Wang, Yidi Zhang, Lijun Liu, Yanmin Zhou

**Affiliations:** 1 Jilin Provincial Key Laboratory of Tooth Development and Bone Remodeling, Hospital of Stomatology, Jilin University, Changchun, 130021 China; 2 Department of Oral Implantology, Hospital of Stomatology, Jilin University, Changchun, 130021 China; 3 School of Basic Medical Sciences, Baicheng Medical College, Baicheng, 137000 China

**Keywords:** Microbiology techniques, Nanobiotechnology, Applied microbiology, Fungi

## Abstract

Due to the increasing demand for eco-friendly, cost-effective and safe technologies, biosynthetic metal nanoparticles have attracted worldwide attention. In this study, silver nanoparticles (AgNPs) were extracellularly biosynthesized using the culture supernatants of *Aspergillus sydowii*. During synthesis, color change was preliminarily judge of the generation of AgNPs, and the UV absorption peak at 420 nm further confirms the production of AgNPs. Transmission electron microscopy and X-ray diffraction were also used to identify the AgNPs. The results shows that AgNPs has crystalline cubic feature and is a polydisperse spherical particle with size between 1 and 24 nm. Three main synthesis factors (temperature, pH and substrate concentration) were optimized, the best synthesis conditions were as follows 50 °C, 8.0 and 1.5 mM. In the biological application of AgNPs, it shows effective antifungal activity against many clinical pathogenic fungi and antiproliferative activity to HeLa cells and MCF-7 cells in vitro. Our research finds a new path to biosynthesis of AgNPs in an eco-friendly manner, and bring opportunity for biomedical applications in clinic.

Nanotechnology is a rapidly developing field today, which has many influences on human life^1,2^. Nanoparticles (NPs) are clusters of atoms in the size range of 1–100 nm with novel properties such as optical effect, quantum size effect, and surface effect. These properties depend on their shape, morphology and size which enable them to interact with microorganisms, plants and animals^3^. As an important metal, silver nanoparticles (AgNPs) have many applications: catalysis, electronics, medical diagnosis and antimicrobial activities^4–6^, and also has other novel activities as anticoagulant, andiabetic and thrombolytic^7–10^.

Methods of synthesizing AgNPs include physical, chemistry, and biological. Physical or chemistry methods require extreme conditions, such as large number of toxic chemicals, high pressure and high temperature, which will have an impact on the environment or require complex equipment^11^. Therefore, it is necessary to develop an environment friendly method to synthesize AgNPs using gentle techniques and nontoxic chemicals during the synthesis process^12,13^. Recently, application of biomaterials for AgNPs synthesis have attracted the attention of investigators, the biomaterials include plant, fungi, bacteria and algae, metabolites of arthropods, enzymes animal and agricultural wastes materials et al.^14–23^.

Fungi can produce large amount of metabolites compared to other microorganisms, making it more suitable for nanoparticles production^24,25^. Many articles have reported that fungi have the ability to synthesize nanoparticles^26,27^. *Candida albicans* synthesized CdSe nanoparticles was the first report that fungi can be utilized to biosynthesize nanoparticles in 1989^28^. Since then, more and more fungi^29–32^ have been reported to have the ability to synthesize nanoparticles, such as *Trichoderma asperellum, Cladosporium cladosporioides*, *Fusarium* spp. and *Aspergillus* spp*.*^33–35^. Among these fungi, filamentous fungi have attracted people's attention because the AgNPs produced by filamentous fungi have good morphological characteristic and the particles are stable, this can make AgNPs have a wide range of applications^14^.

In this study, we report the extracellular biosynthesis of AgNPs by *Aspergillus sydowii*. The characteristics of synthesized AgNPs were identified by X-ray diffraction (XRD), ultraviolet–visible spectroscopy and transmission electron microscopy (TEM). We optimized the key factors in the synthesis process of AgNPs and discussed the effects of different conditions. The antifungal and antiproliferative activities of AgNPs were also tested. This is the first report that *Aspergillus sydowii* can biosynthesis AgNPs using extracelluar supernatants in an environment friendly manner^36^. And the biosynthesized AgNPs has excellent biological application functions.

## Results and discussion

### Strain identification

**Figure 1:**
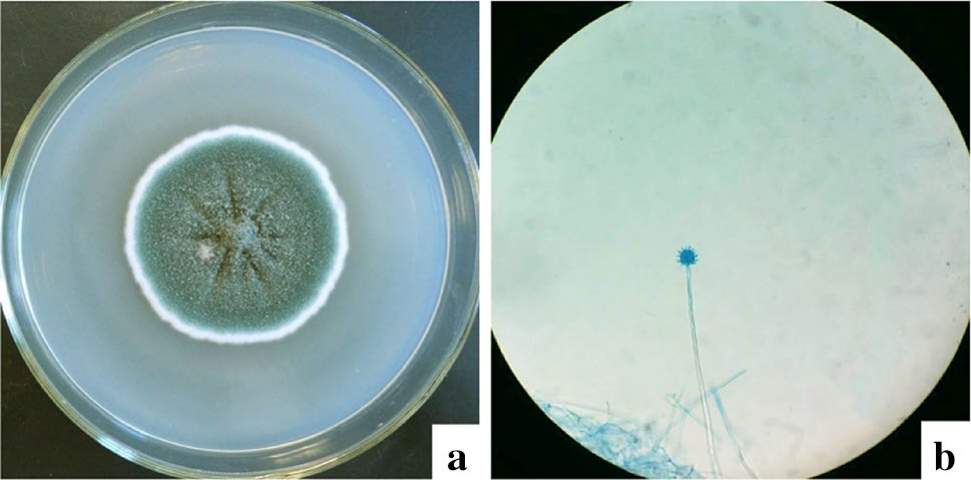
Morphology of *Aspergillus sydowii*. (**a**) Macroscopic morphology (28 ℃, 7d); (**b**) microscopic morphology (× 400).

Nanotechnology is developing rapidly in the field of biological science. In this study, AgNPs were successfully synthesized by *Aspergillus sydowii* cell filtrate. The strain was isolated from soil and cultured on SDA medium at 28 °C. Based on the characteristics of colony morphology (the peripheral hyphae are white, there are a large number of spores deposit in the middle of the colony) (Fig. 1a) and micromorphology (the hyphae contain conidiophores and sporangia) (Fig. 1b), the strain was identified as a member of Aspergillus genus.

**Figure 2:**
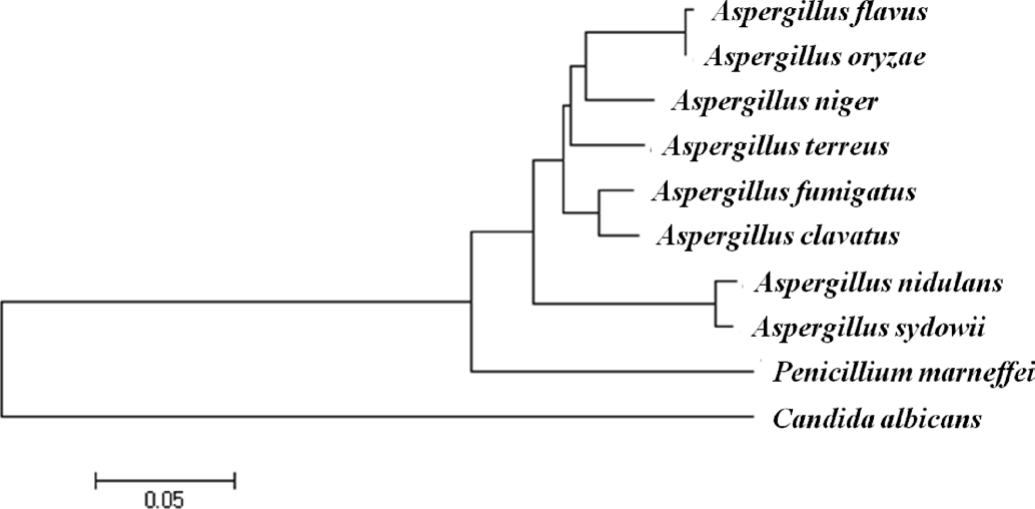
Phylogenetic tree was created using ITS sequence.

In addition to morphological identification, molecular identification was also applied. The ITS nucleotide sequence is 100% homology to *Aspergillus sydowii* (accession number NR_131259.1) according to the GenBank database. A phylogenetic tree was created by MEGA 5.2 with ITS nucleotide sequence to check homologues from different fungi (Fig. 2). According to morphological and molecular characteristics, the strain was identified as *Aspergillus sydowii*. Many *Aspergillus* species have been reported to be able to synthesize silver nanoparticles, due to their ability to produce abundant secondary metabolites^29–31,34,37^. This is the first report of the synthesis of AgNPs by *Aspergillus sydowii* using extracellular supernatant in an eco-friendly manner.

### AgNPs biosynthesis and characterization

**Figure 3:**
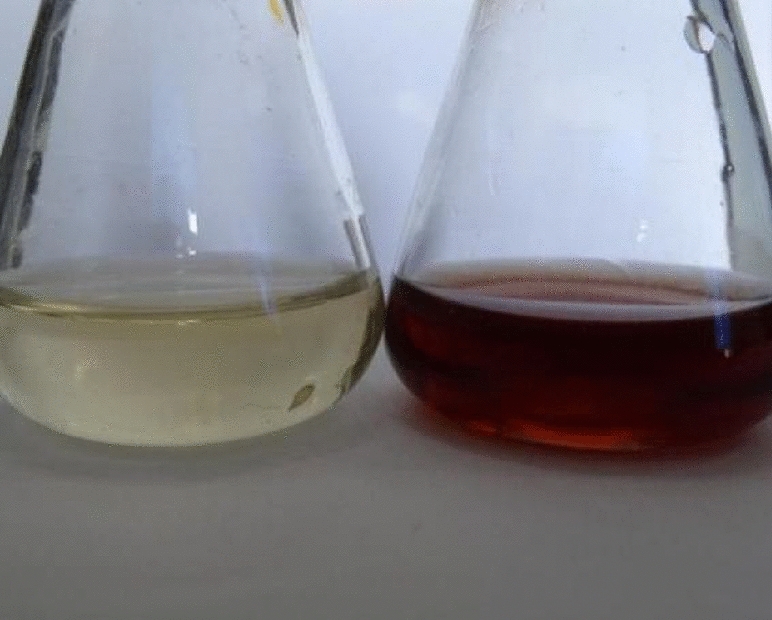
The cell filtrate of *Aspergillus sydowii* without AgNO_3_ (left) and with AgNO_3_.

AgNPs was carried out in dark at room temperature, when AgNO_3_ was added to the *Aspergillus sydowii* cell filtrate, the color changed from yellow to brown(AgNO_3_ formed) (Fig. 3). This phenomenon is due to the surface plasmon resonance of AgNPs.

**Figure 4:**
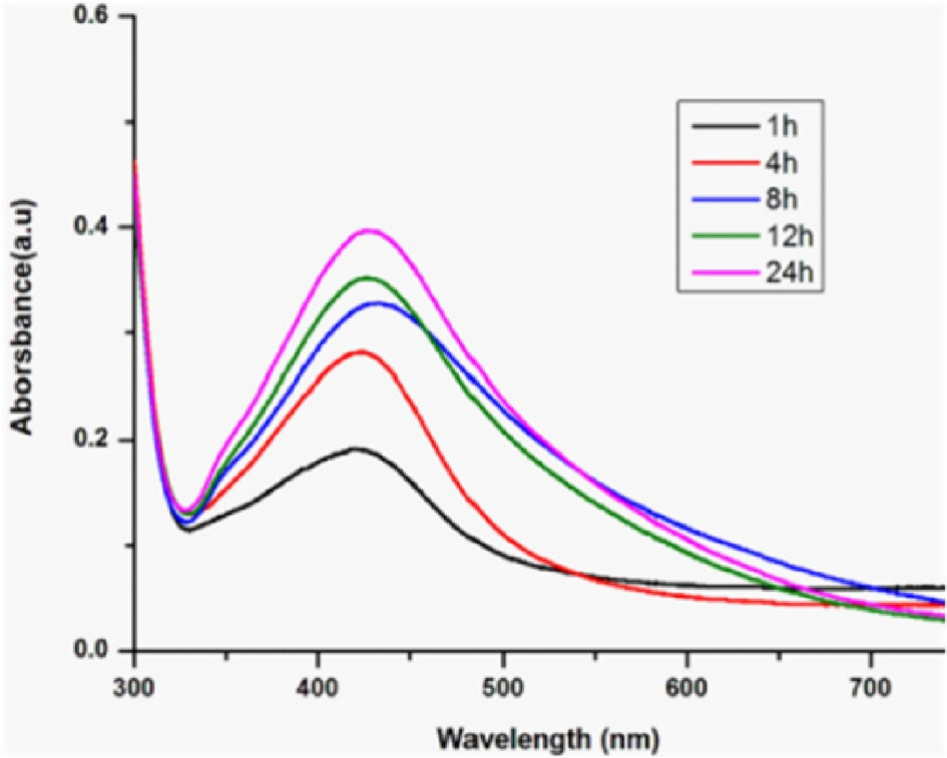
UV–visible absorption spectrum of AgNPs synthesized by *Aspergillus sydowii* with different time.

Different reaction time (1–24 h) of synthesis process was detected and monitored by ultraviolet–visible spectrophotometer with scanning spectrum ranging from 300 to 700 nm. As shown in Fig. 4, a strong absorbance peak at 420 nm was detected, which indicated the existence of AgNPs^38^. From the UV results we can observe that as the time of reaction increases, the maximum absorption peak at 420 nm also increased. This indicated that the amount of synthesized AgNPs is increasing gradually in a time-dependent manner^39^. Meanwhile, the UV absorption peaks at 420 nm in the 1st hour and 24 th hour are similar, which indicates that the AgNPs are well dispersed without aggregation^37,40^.

**Figure 5:**
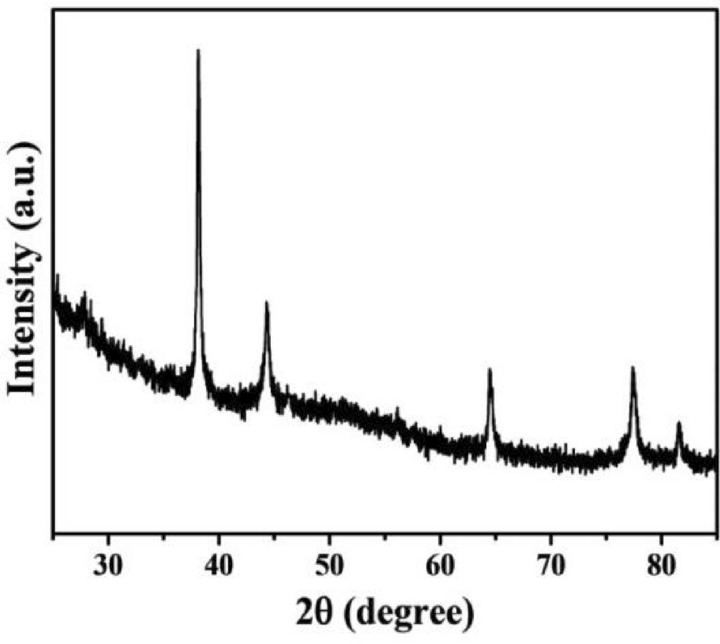
X-ray diffraction patterns of synthesized AgNPs.

In this research, the crystalline feature of AgNPs was studied by XRD. As shown in Fig. 5, four diffraction peaks were exhibited in (111), (200), (220), (311) lattice planes of the whole spectrum at 2*θ* value of 38.18°, 44.42°, 64.62° and 77.78°, respectively. This was identical to silver cubic structure with the standard diffraction pattern (JCPDS File No. 04-0783).

**Figure 6:**
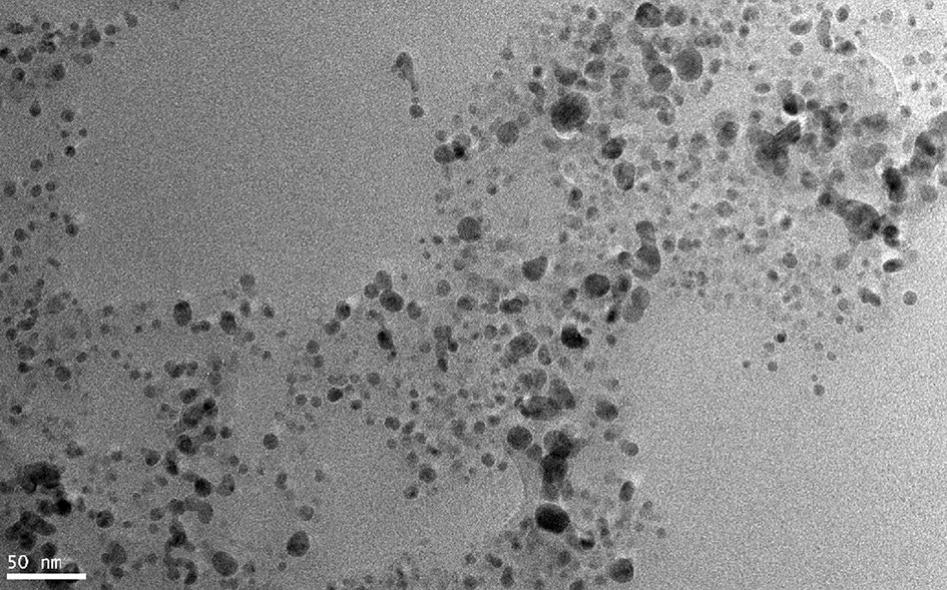
The micrograph of AgNPs synthesized by reduction of silver nitrate with the cell filtrate of *Aspergillus sywdoii*.

**Figure 7:**
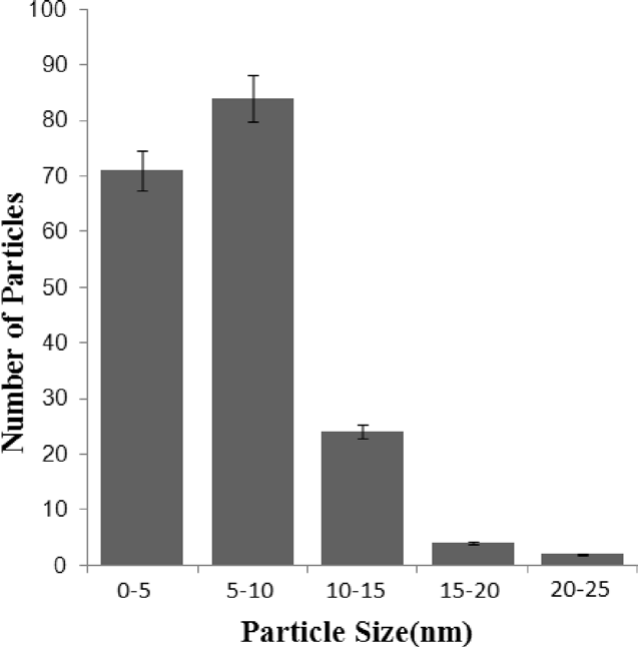
Particle size distribution of AgNPs.

TEM was used to observe the shape and size of synthesized AgNPs. As shown in Fig. 6, the shapes of AgNPs are spherical or close to spherical. The average size of AgNPs were 12 ± 2 nm, Fig. 7 showed that the synthesized particle diameters ranging from 1 to 21 nm. 38% of the AgNPs are between 0 and 5 nm in diameter, 45% of the AgNPs are between 5 and 10 nm in diameter and 12% of the AgNPs are between 10 and 15 nm in diameter. Through the results of XRD and TEM, we found that AgNPs have good morphological characteristics, the appearance of AgNPs were spherical or close to spherical, and the particle size is small and uniform.

### Optimization synthesis procedure

**Figure 8:**
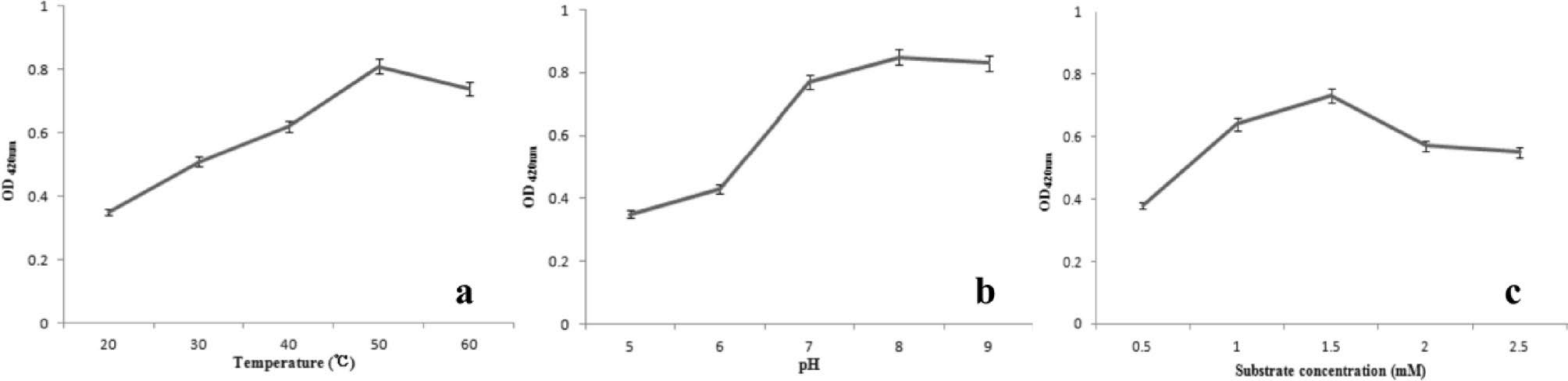
Optimization of reaction conditions of AgNPs. (**a**) Time on AgNPs biosynthesis by cell filtrate of *Aspergillus sywdoii*; (**b**) pH; (**c**) substrate concentration.

In this study, we optimized three main parameters of AgNPs synthesis process. As shown in Fig. 8a, we investigated the different temperature (20–60 ℃) effect on AgNPs production. When the temperature increases from 20 to 50 ℃, the absorption peak also increases, but decreases at 60 °C. The optimal synthesis temperature is 50 °C.

Different strains require different pH environments to synthesize nano silver, in this study we investigated the effect of PH on the synthesis of AgNPs. The results shows that absorbance is increased when pH increases from 5.0 to 8.0 (Fig. 8b). This means the optimal pH is 8.0.

Finally, we analyzed the effect of different AgNO_3_ concentrations on silver nanoparticles production. Figure 8c shows that the optimal substrate concentration was 1.5 mM. This suggest that the synthesis conditions should to be maintained at the appropriate concentration.

The above results indicate that there are different influencing factors during the synthesis of AgNPs, which need to optimized to achieve the best synthesis state. Therefore, the best conditions for biosynthesis of AgNPs are 50 °C, pH 8.0 and 1.5 mM substrate concentration, respectively.

### AgNPs antifungal activity

In recent years, cases of fungal infections have become more common, but the available pools of antifungal drugs are relatively few. In this research, twelve fungal pathogens were selected to test the antifungal activity of AgNPs, including *Aspergillus* spp., *Fusarium* spp., *Candida* spp. *Cryptococcus neoformans* and *Sporothrix schenckii*. Table 1 shows the MIC values of the tested strains. Compared with itraconzole and fluconazole, the AgNPs has wider antifungal range. It showed good antifungal activities to all tested fungus strains in a relatively low concentration.

**Table 1:** Antifungal activity of Itraconazole, Fluconazole and AgNPs.

Tested fungal strains	MIC (μg/mL)		
	Itraconazole	Fluconazole	AgNPs
*Candida albicans*	0.03	0.25	0.25
*Candida glabrata*	0.25	8	0.125
*Candida parapsilosis*	0.25	8	0.25
*Candida tropicalis*	0.25	0.25	0.125
*Fusarium solani*	> 16	> 64	1
*Fusarium moniliforme*	> 16	> 64	2
*Fusarium oxysporum*	> 16	> 64	4
*Aspergillus flavus*	0.125	> 64	1
*Aspergillus fumigatus*	0.03	> 64	2
*Aspergillus terrus*	0.25	> 64	2
*Sporothrix schenckii*	0.125	> 64	0.25
*Cryptococcus neoformans*	0.0625	2	0.25

**Figure 9:**
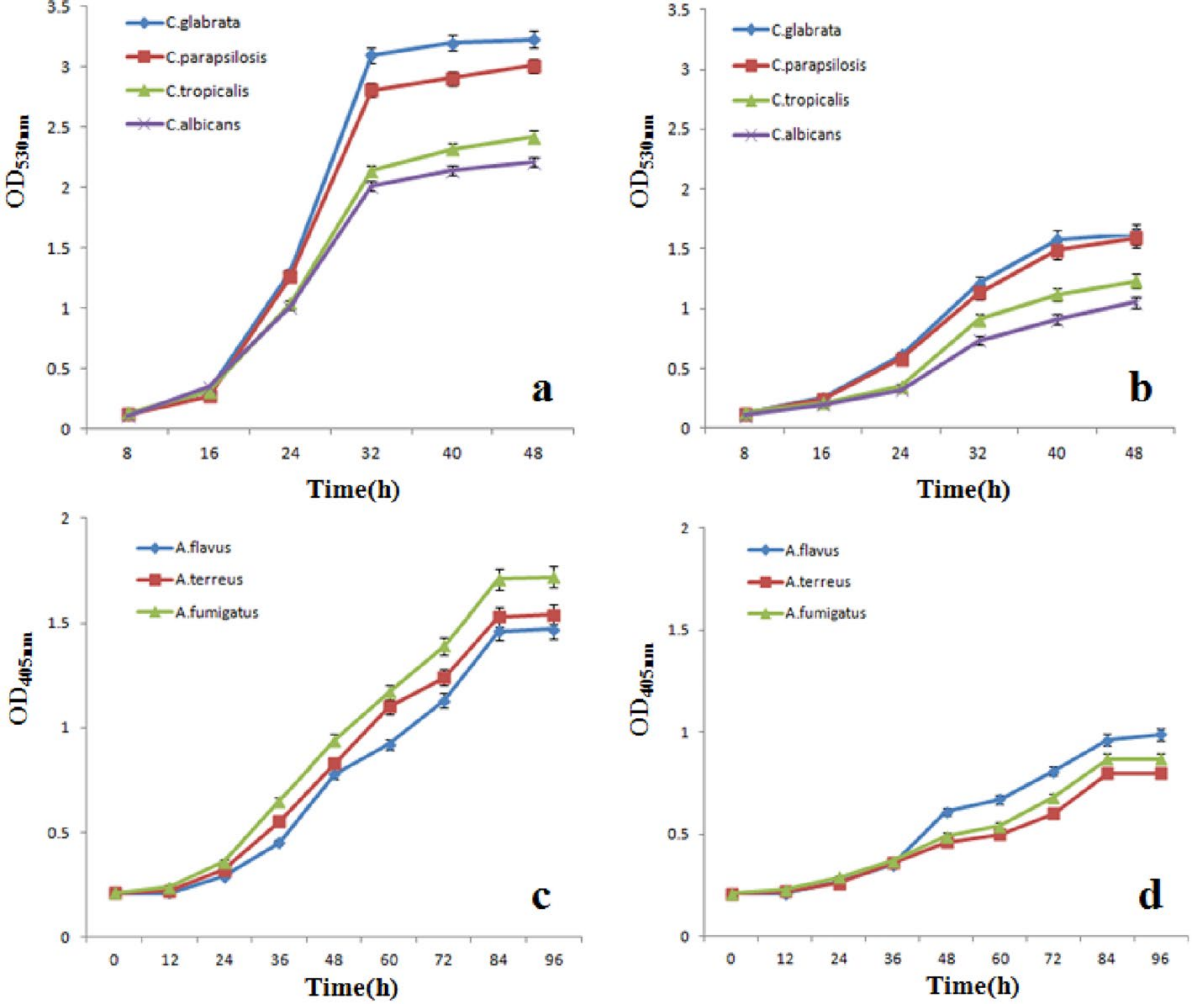
Fungal growth kinetics of *Candida* and *Aspergillus* in the absence of AgNPs (**a**,**c**) and the presence of AgNPs (**b**,**d**) (*p* < 0.05).

In clinical systemic fungal infection cases, yeast and filamentous fungi occupied most of them. In our research, we further investigated the growth curve of yeast and filamentous fungi with or without AgNPs. Figure 9 shows the growth curve of *Candida* (*C. albican*, *C. glabrata, C. parapsilosis* and *C. tropicalis*) and *Aspergillus* (*A. fumigatus*, *A. flavus*, and *A.terreus*). The growth curve of *Candida *spp. or *Aspergillus* spp. were significantly reduced when medium contains AgNPs.

The results of antifungal test indicated that AgNPs have the potential to become an effective antifungal agent in future. Of course, more antifungal mechanisms need to be investigated before clinical application. At present, there have been some mechanism studies. Dibro et al.^41^ stated that the diameter of silver nanoparticles is very small (10–100 nm) which can easily penetrate the cell wall to reach the cell membrane and enter the cell with its unique size effect and surface effect. Due to the large surface area of AgNPs, it’s easy to react in cell solution: Ag = Ag^+^  + e^−^. After entering the pathogen cell, the silver ion quickly binds to the enzyme protein sulfhydryl (–SH), deactivating some of the necessary enzymes, causing cell lose the ability to divide and reproduce. According to Feng’s research^42^, silver ions can combine with DNA bases in pathogenic fungi to form cross-links, and then replace the hydrogen bonds adjacent to nitrogen in purines and pyrimidines. It will change the structure of fungal DNA and lose the ability to replicate so as to achieve the effect of killing fungi. In addition, in Kim’s^43^ opinion the combination of AgNPs and protein molecules on the fungi surface results in denaturation reaction, cleavage the proton pump, and enhance the permeability of the membrane protein or phospholipid bilayer. The leakage of H^+^ leading to the lysis of fungal cell membrane causing fungal cell damage.

### AgNPs antiproliferative activity

**Figure 10:**
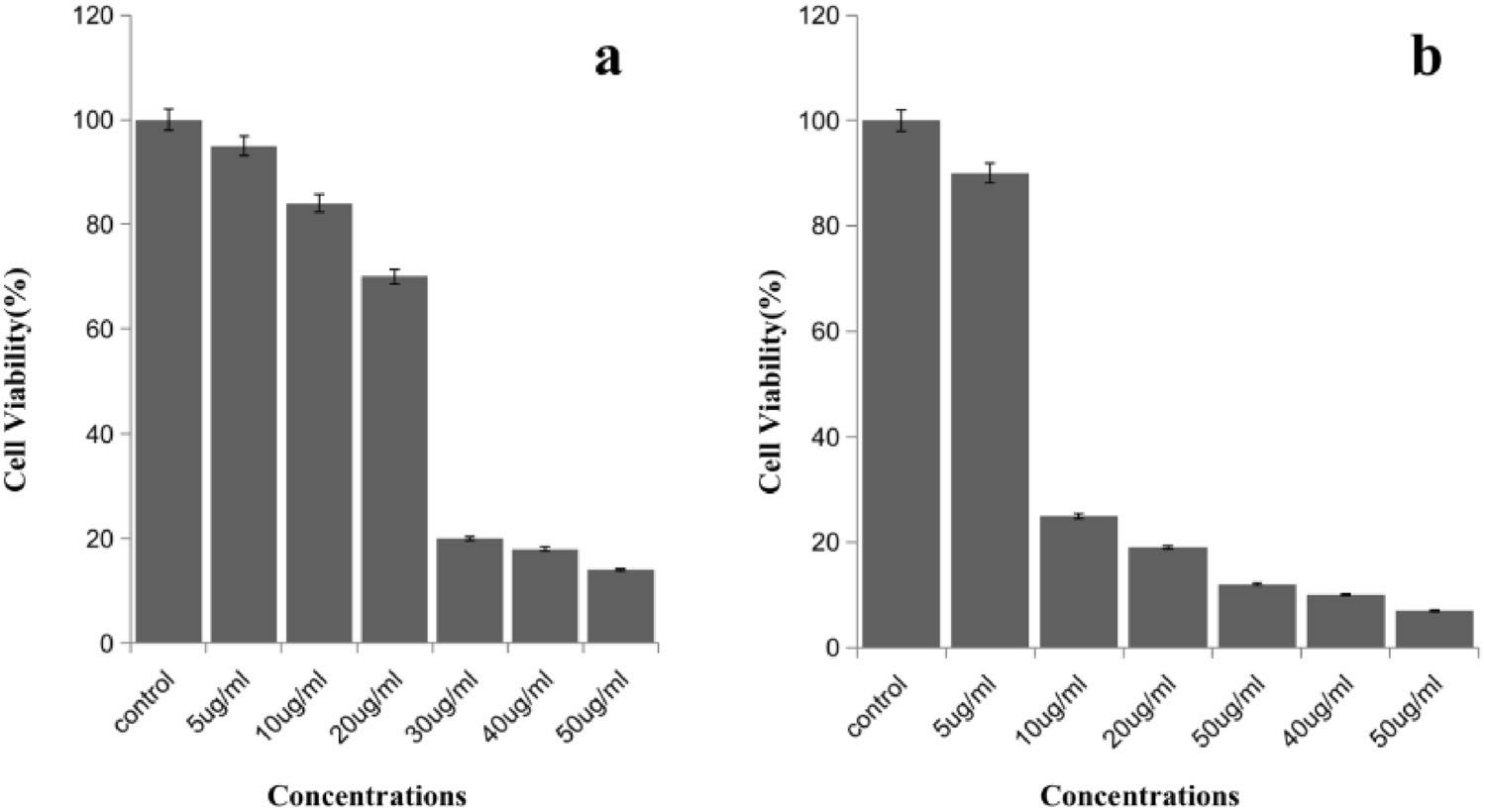
MTT assay in HeLa cells (**a**) and MCF-7 cells (**b**) following the exposure of various concentrations of AgNPs for 24 h (*p* < 0.05).

Besides antifungal test, the antiproliferative ability of AgNPs also aroused our attention in this research. Cancer is a dangerous, hard-to-cure and world-wide distributed disease. It always needs new and effective agents to control this disease. In this study we made antiproliferative test. The MTT results shows that the antiproliferative activity of AgNPs to HeLa cells and MCF-7 cells is in a concentrations dependent manner as shown in Fig. 10. When AgNPs’ concentration exceed 30 μg/mL or 10 μg/mL, it significantly inhibit HeLa and MCF-7 tumor cell growth respectively, showed good antiproliferative activity. There are some studies have proved that AgNPs have antiproliferative properties^44–46^. Due to their surface charges and sizes, the mechanism of antiproliferative activity mainly focuses on the following points: (1) inducing oxidative stress; (2) damaging DNA; (3) damaging mitochondria; (4) activating immune system; (5) inducing cell cycle arrest; (6) activating apoptosis.

## Conclusion

In this study, AgNPs were first time biosynthesized by *Aspergillus sydowii* cell filtrate, the synthesis process was quite gentle and without toxic agents. The AgNPs shows good stability and uniformity. In the biological application of AgNPs, we found the AgNPs have significant antifungal activity against pathogen fungi. Meanwhile, AgNPs also have good antiproliferative activity against HeLa and MCF-7 tumor cells. The capability to synthesize AgNPs using *Aspergillus sydowii* is promising as an eco-freindly, simple, and sustainable method of nano-metals, and provide a new strategy for biomedical application.

## Materials and methods

### Strains and materials

Twelve fungi were tested for antifungal activity including: *Candida albicans* ATCC 90028, *Candida glabrata* ATCC 66032, *Candida parapsilosis* ATCC 22019, *Candida tropicalis* ATCC 9928, *Fusarium solani* ATCC 36031, *Fusarium moniliforme* JLCC 31463, *Fusarium oxysporum* JLCC 30866, *Aspergillus flavus* IFM 55648, *Aspergillus fumigatus* IFM 40808, *Aspergillus terrus* JLCC 30844, *Sporothrix schenckii* JLCC 32757, and *Cryptococcus neoformans* ATCC 36556 (American Type Culture Collection, ATCC; Culture Collection of Jilin University, Mycology Research Center, JLCC; Institute for Food Microbiology, Medical Mycology Research Center, Chiba University, IFM). Cervical cancer cells (HeLa, ATCC CCL-2) and breast cancer cells (MCF-7, ATCC CRL-3435) were purchased from ATCC. Sabouraud medium was purchased from BD (Becton, Dickinson and company); the silver nitrate (AgNO_3_) used in this study was purchased from Sigma-Aldrich. 3-(4,5-dimethylthiazol-2-yl)-2,5-diphenyltetrazolium bromide (MTT), RPMI 1640, and fetal bovine serum (FBS) were purchased from Invitrogen.

### Morphology and molecular identification

*Aspergillus sydowii* used for AgNPs synthesis in this study was isolated from soil, maintained on sabouraud’s dextrose agar (SDA) medium at 28 °C and stored at 4 °C. The fungus was identified by colony morphological and micromorphological characteristics, also identified by molecular method using Internal Transcribed Spacer (ITS) gene sequence. Strain was grown on a SDA medium at 28 °C for 10 days to study its colony morphology. The micromorphological characteristics of the strain were observed by slide culture method. Cultures were incubated on slides of SDA at 28 °C for 5 days, the slide was stained with lactophenol cotton blue and then observed through a light microscope.

The fungus molecular identification was carried out as described previously^37^. The fungus was incubated into 50 mL sabouraud medium at 28 °C for 3 days with rotary, collected the biomass by centrifugal. Genomic DNA of the fungus was isolated using a TAKARA DNA extracted kits. PCR were applied to amplify the internal transcribed spacer region of the fungus. Homology research was performed in NCBI website using BLASTn.

### Biosynthesis of AgNPs

*Aspergillus sydowii* was grown in sabouraud medium, which was inoculated with 1 × 10^6^ spores in 250 mL flasks. Flasks were incubated at 28 ± 2 °C in a rotary shaker (140 rpm) for 5 days. Fungal biomass were harvested by filtration through Whatman filter paper, and washed with double distilled water to prevent contamination from medium. 20 g (wet weight) biomass was placed in a flask containing 100 mL double distilled water, incubated at 28 °C for 24 h, the suspension was filterd. AgNO_3_ solution was added in a 200 mL flask mixed with 50 mL fungal filtrate, wherein the ratio of AgNO_3_ to cell filtrate was 9:1. Then the flask was incubated as previous for 48 h.

### Characterization of silver nanoparticles

Color change of cell filtrate and AgNO_3_ solution mixture was the first indicator of detection for AgNPs. 3 mL of different reaction time samples were monitored by ultraviolet–visible spectrophotometer (UV-2450; Shimadzu, Tokyo, Japan) scanning from 300 to 700 nm with 1 nm resolution.

After incubation, AgNPs were collected by centrifugation at 12,000*g* for 10 min and washed three times with deionized water. AgNPs were further identified by X-ray diffraction (XRD) and transmission electron microscopy (TEM). Crystal structure of AgNPs were tested by XRD (RINT 2000 vertical goniometer) operating at 200 mA and 50 kV with Cu Kα radiation (λ = 1.5405 Å). Characterization of morphology, distribution and size of AgNPs were detected by TEM (Tecnai F20 S-Twin) at 200 kV. And the AgNPs sizes were tested by Debye–Scherrer method^33^.

### Optimization of AgNPs biosynthesis

Effect of three main parameters (temperature, pH and substrate concentration) on the AgNPs production were optimized by changing only one factor at a time, such as temperature (20, 30, 40, 50 and 60 °C), pH (5, 6, 7, 8, and 9) and substrate concentration (0.5, 1.0, 1.5, 2.0, and 2.5 mM AgNO_3_). The absorbance of sample was measured at 420 nm using UV spectrophotometer.

### Antifungal activity of AgNPs

The antifungal activity of AgNPs synthesized by *Aspergillus sydowii* were tested against several common clinical fungi, including *Candida albicans*, *Candida glabrata*, *Candida parapsilosis*, *Candida tropicalis*, *Fusarium solani*, *Fusarium moniliforme*, *Fusarium oxysporum*, *Aspergillus flavus*, *Aspergillus fumigatus*, *Aspergillus terreus*, *Sporothrix schenckii*, and *Cryptococcus neoformans*, the antifungal method was based on CLSI (Clinical and Laboratory Standards Institute) guidelines (document M38-A2). In brief, AgNPs were mixed with RPMI 1640 medium with 2% glucose, and the final concentrations were adjusted ranging from 0.125 to 64 μg/mL. The spore suspensions of tested strains were adjusted to 2 × 10^4^ CFU/mL. The AgNPs and spores suspension were mixed and added in 96-well plates, incubation at 37 °C or 28 °C for yeast and filamentous fungi respectively. The plates were assessed 24 h or 48 h after incubatation. MICs (minimal inhibitory concentrations) were defined as the lowest concentration of AgNPs that inhibited the fungal growth by 90%. All tests were repeated at least three times.

### Fungal growth kinetics

AgNPs fungal growth kinetics effect was tested for Yeast and filamentous fungi (*C*. *albicans*, *C*. *glabrata*, *C*. *parapsilosis*, *C*. *tropicalis*, *A. fumigatus*, *A. flavus*, and *A.terreus*). Sabouraud medium containing 2.5 μg/mL AgNPs was inoculated with fungal cells (1 × 10^6^ CFU/mL). 150 mL flask supplemented with 30 mL mixture was incubated at 37 °C for 36 h on rotary shaker; the growth curves of *Candida* spp*.* and *Aspergillus* spp*.* were graphed using sequential OD measurements. All assays were carried out three times.

### Antiproliferative assay

Antiproliferative activity of AgNPs was examined by using standard MTT assay as previously^47^. In brief, cells (1 × 10^4^ cells/well) were plated in 96 well plates incubated at 37 °C, in 5% CO_2_ atmosphere for 24 h. Formed monolayer of cells were treated with various concentrations (1, 2, 5, 10, 25, and 50 μg/mL) of AgNPs. After 24 h incubation, MTT was added in each well and incubated for 4 h further. At last, 200 μL/well DMSO was added to each well and mixed in a gentle manner. Then the absorbance of each well was measured at 570 nm; and the cell viability was calculated as percentage of control.

### Statistics analysis

The data of this research were shown as means ± SD. The figures represented 3 separate tests. Variance analysis were used to estimate the differences among the groups. *p* < 0.05 was considered statistically significant.

### Ethical approval

This article does not contain any studies involving animals performed by any of the authors.
